# Validity of pediatric index of mortality 2 (PIM2) score in pediatric acute liver failure

**DOI:** 10.1186/s13054-014-0665-z

**Published:** 2014-12-02

**Authors:** Claire Elizabeth Matthews, Chulananda Goonasekera, Anil Dhawan, Akash Deep

**Affiliations:** St Thomas’ Hospital, Westminster Bridge Road, London, SE1 7EH UK; King’s College Hospital, Denmark Hill, London, SE5 9RS UK

## Introduction

Pediatric acute liver failure (PALF) is an aggressive clinical syndrome in which a previously healthy child rapidly loses hepatic function and can become critically ill within days [1,2]. The mortality rate has been reported as high as 70% without a liver transplant, which is the only curative treatment to date [3]. However, when the current criterion of an international normalized ratio of more than 4 for listing for transplantation is used, 10% who are listed spontaneously recover and another 10% die while waiting to be listed [4]. This has serious implications for scarce donor organs and the risk of subjecting a child to unnecessary transplantation, surgical complications, and lifelong immunosuppression. The underlying challenge is to identify early enough those with PALF for whom chances of survival without a transplant are null, so that these can be super-urgently listed. As such, early mortality prediction is as important as the criteria for listing for transplantation.

The goal of the present study was to investigate the prognostic accuracy of the commonly used mortality prediction score known as pediatric index of mortality 2 (PIM2). PIM2 uses 10 variables on admission to the pediatric intensive care unit (PICU) to assess risk of mortality and in the UK is used as a measure of the quality of service. All children who were admitted to the PICU at King’s College Hospital between 2003 and 2013 and who had PALF as defined by the PALF study group [1] were entered into a database which records routinely collected data that included daily clinical, biochemical, and treatment variables. We compared PIM2 scores on admission to 28-day survival with or without transplantation. As this was a retrospective analysis of routinely collected anonymized data, ethical approval or informed consent was not required. This study has been registered as a service evaluation project at King’s College Hospital.

## Findings

One hundred and three children (55 males; median age 2 years, age range 0 to 19 years) were included. Forty-three received a liver transplant; of these, five died (mortality of 11.6%). Of the 60 who were not transplanted, 20 died (mortality of 33.3%). A receiver operating characteristic (ROC) curve was developed by using the study population and their outcomes. An area under the curve (AUC) of more than 0.75 was considered clinically useful [5]. The AUC in transplanted children was 0.346 (95% confidence interval (CI) 0.000 to 0.671), suggesting that PIM2 is of absolutely no value in predicting mortality in transplanted children. In the non-transplanted group, the AUC of ROC was 0.712 (95% CI 0.561 to 0.864), which was not sensitive or specific enough to minimize the risk of wrong categorization for transplantation.

Thus, we conclude that PIM2 is not fit for use in PALF as it neither predicts mortality nor identifies children in need of liver transplantation. It may also bias the quality assessment of PICUs in liver centers. As such, the quest for a better predictive score in PALF continues.

## Abbreviations

AUC: Area under the curve
CI: Confidence interval
PALF: Pediatric acute liver failure
PICU: Pediatric intensive care unit
PIM2: Pediatric index of mortality 2
ROC: Receiver operating characteristic
